# Connective tissue growth factor promoter activity in normal and wounded skin

**DOI:** 10.1186/1755-1536-1-3

**Published:** 2008-10-13

**Authors:** Mohit Kapoor, Shangxi Liu, Kun Huh, Sunil Parapuram, Laura Kennedy, Andrew Leask

**Affiliations:** 1 CIHR Group in Skeletal Development and Remodeling, Division of Oral Biology and Department of Physiology and Pharmacology, Schulich School of Medicine and Dentistry, Dental Sciences Building, University of Western Ontario, London, Ontario, N6A 5C1, Canada

## Abstract

In skin, connective tissue growth factor (CTGF/CCN2) is induced during tissue repair. However, what the exact cell types are that express CTGF in normal and wounded skin remain controversial. In this report, we use transgenic knock-in mice in which the Pacific jellyfish *Aequorea victoria *enhanced green fluorescent protein (E-GFP) gene has been inserted between the endogenous CTGF promoter and gene. Unwounded (day 0) and wounded (days 3 and 7) skin was examined for GFP to detect cells in which the CTGF promoter was active, α-smooth muscle actin (α-SMA) to detect myofibroblasts, and NG2 expression to detect pericytes. In unwounded mice, CTGF expression was absent in epidermis and was present in a few cells in the dermis. Upon wounding, CTGF expression was induced in the dermis. Double immunolabeling revealed that CTGF-expressing cells also expressed α-SMA, indicating the CTGF was expressed in myofibroblasts. A subset (~30%) of myofibroblasts were also NG2 positive, indicating that pericytes significantly contributed to the number of myofibroblasts in the wound. Pericytes also expressed CTGF. Collectively, these results indicate that CTGF expression in skin correlates with myofibroblast induction, and that CTGF-expressing pericytes are significant contributors to myofibroblast activity during cutaneous tissue repair.

## Background

Tissue repair involves the reconstitution of connective tissue. During this process, fibroblasts migrate into the wound where they produce and subsequently remodel extracellular matrix (ECM), resulting in wound closure. These events are mediated by a specialized form of fibroblasts, termed myofibroblasts [1]. Persistence of the myofibroblast is a hallmark of fibrotic lesions [2,3]. However, the origin of myofibroblasts during tissue repair remains controversial [4]. For example, it is uncertain to what extent activated myofibroblasts derive from local recruitment of fibroblasts or from pericytes surrounding blood vessels [5].

Connective tissue growth factor (CTGF/CCN2), a member of the CCN family of proteins [6], acts through integrins and heparan sulfate-containing proteoglycans (HSPGs), which are the *bona fide *CTGF receptors, to directly induce and modify adhesive signaling both independently and in response to growth factors and extracellular matrix [7-10]. CTGF is expressed in mesenchymal cells during development and wound healing [11,12] and is overexpressed in fibrotic diseases [13]. Studies on CTGF gene regulation have been performed using a variety of cell culture systems, including fibroblasts, and mesangial and cancer cells [13-17]. However, what the actual cell types that express CTGF *in vivo *are remains somewhat controversial. For example, studies employing various antibodies directed toward CTGF have been used to examine CTGF protein expression in development, with widely divergent results (see [18,19]); one study used Western blot analysis of tissue extracted from wound chambers introduced subcutaneously to show that CTGF is induced in tissue repair, but is absent normally [11]. Conversely, another study used *in situ *hybridization to show that CTGF mRNA was constitutively expressed in normal human skin [20]. These problems may have arisen due to possible difficulties regarding either the sensitivity of the detection methods used or cross-reactivity among related family members. Moreover, the CCN family consists of secreted proteins, making the identification of individual cell types expressing CTGF difficult. Careful *in vivo *analysis of cells expressing CTGF has not been performed.

The green fluorescent protein (GFP) reporter has been used *in vivo *to detect the specific cells in which gene promoters are active [21,22]. Such a reporter is particularly useful to avoid possible issues regarding sensitivity of *in situ *hybridization to particular mRNAs, especially regarding possible cross-hybridization among members of related protein families, and when there are possible issues regarding antibody sensitivities. In this study, we report the findings from our investigation of knock-in mice where enhanced GFP (E-GFP) has been inserted between the endogenous CTGF promoter and gene. These mice were derived from a previously reported screen in which bacterial artificial chromosomes (BACs) were inserted into the genome [23]. Mice were subjected to the dermal punch model of tissue repair, and the expression of CTGF was detected using an anti-GFP antibody. Moreover, activated myofibroblasts and pericytes were detected using appropriate markers. Our data provide the first careful analysis of the cell types that express CTGF in skin. Moreover, our data provide new insights into the origin of the activated fibroblasts during normal tissue repair.

## Results

### CTGF promoter activity is induced post-wounding in myofibroblasts

**Figure 1:**
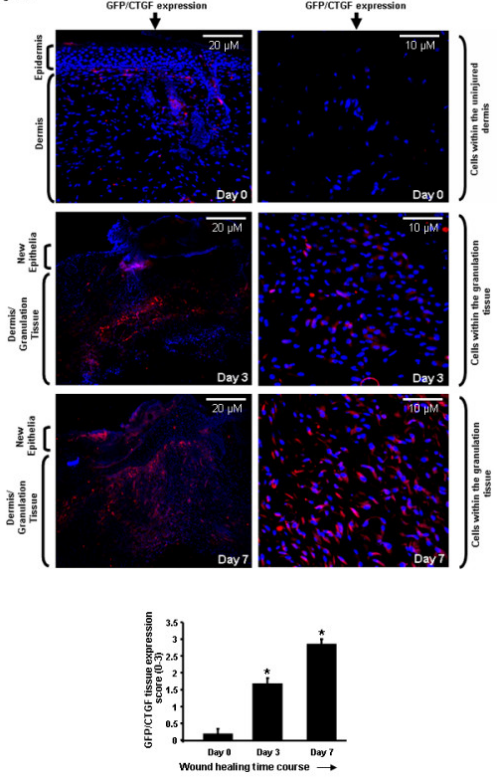
**Connective tissue growth factor (CTGF) promoter activity in skin pre- and post-wounding**. Skin of CTGF/enhanced green fluorescent protein (E-GFP) hemizygous mice (pre-wounding, day 0, or post-wounding, day 3 or day 7) was fixed, sectioned, and stained with 4',6-diamidino-2-phenylindole (DAPI) to detect nuclei or anti-GFP antibody to detect cells in which the CTGF promoter was active (lefthand panels, 10 × magnification of dermal tissue; righthand panels, 40 × magnification of dermal tissue). Semiquantitative analysis of GFP expression was performed as described in Methods. Note that CTGF promoter activity, as determined by GFP-positive cells, was absent in the epidermis (day 0); however, a small number of dermal cells were positive for GFP expression. Post-wounding, the CTGF promoter was active in granulation tissue. *, significant induction compared to day 0 unwounded skin. Representative data from four wounds from four separate animals per timepoint is shown.

**Figure 2:**
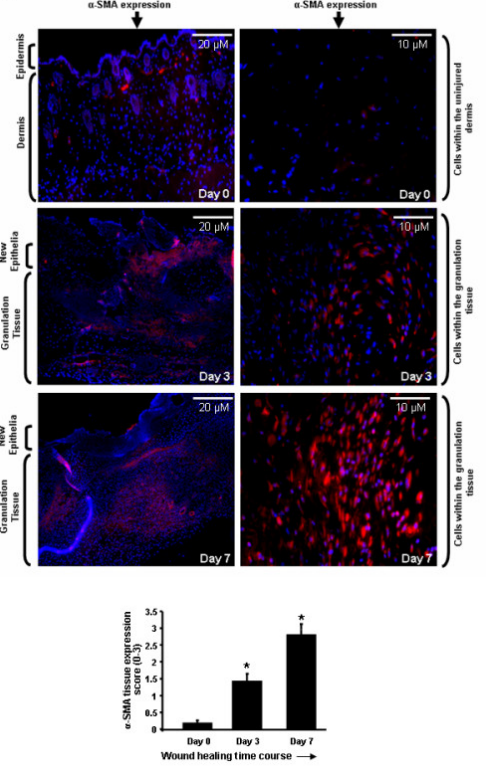
**The presence of α-smooth muscle actin (SMA) expression myofibroblast pre- and post-wounding**. Skin of connective tissue growth factor/enhanced green fluorescent protein (CTGF/E-GFP) hemizygous mice (pre-wounding, day 0, or post-wounding, day 3 or day 7) was fixed, sectioned, and stained with 4',6-diamidino-2-phenylindole (DAPI) to detect nuclei or anti-α-SMA antibody to detect myofibroblasts (lefthand panels, 10 × magnification of dermal tissue; righthand panels, 40 × magnification of dermal tissue). Quantitiation of myofibroblasts was performed as described in Methods. Note that α-SMA expression was absent in the epidermis (day 0); however, a small number of dermal cells were positive for α-SMA expression. Post-wounding, myofibroblasts appeared within granulation tissue. *, significant induction compared to day 0 unwounded skin. Representative data from four wounds from four separate animals per timepoint is shown.

**Figure 3:**
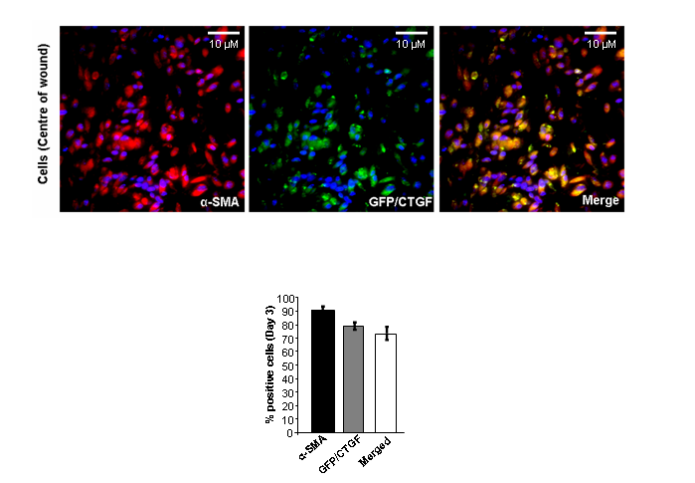
**The connective tissue growth factor (CTGF) promoter is active in myofibroblasts**. Skin of CTGF/enhanced green fluorescent protein (E-GFP) hemizygous mice (post-wounding, day 3) was fixed, sectioned, and stained with 4',6-diamidino-2-phenylindole (DAPI) to detect nuclei, anti-α-smooth muscle actin (SMA) antibody to detect myofibroblasts, and anti-GFP antibody to detect CTGF promoter activity (40 × magnification of dermal tissue). The percentage of fibroblasts within the wound that were α-SMA positive, GFP positive, and both GFP and α-SMA positive was calculated as described in Methods. Note that ~75% of the cells in the wound area were myofibroblasts that expressed GFP. Representative data from four wounds from four separate animals per timepoint is shown.

To date, the cell types that express CTGF in skin pre- and post-wounding have not been known. To address this outstanding issue, we used transgenic mice in which the E-GFP gene has been inserted between the endogenous CTGF promoter and gene. As E-GFP is not secreted, the precise cells expressing it can be detected using an anti-GFP antibody. An anti-GFP antibody was used, as opposed to direct examination of E-GFP, to avoid possible issues due to autofluorescence. When unwounded skin was examined, GFP expression was not detected in the epidermis (Figure 1, day 0). A few GFP-positive cells were detected in the hair follicle and in the dermis; however, GFP-positive cells were essentially absent in unwounded skin (Figure 1, day 0). By 3 days post-wounding, GFP-positive cells appeared in the dermis, but were absent from the epithelia (Figure 1, day 3). By 7 days post-wounding, abundant GFP-positive cells were present in the newly-synthesized tissue (Figure 1, day 7). Similar patterns of expression were observed when tissue sections were stained with anti-α-smooth muscle actin (SMA) antibody to detect activated fibroblasts (that is, the presence of myofibroblasts) (Figure 2). Moreover, cells in which the CTGF promoter was active were also α-SMA positive (Figure 3). Collectively, these data indicate that CTGF promoter activity is minimal in unwounded skin and is induced post-wounding in myofibroblasts.

### Approximately a third of the myofibroblasts in the day 7 wound are pericytes

**Figure 4:**
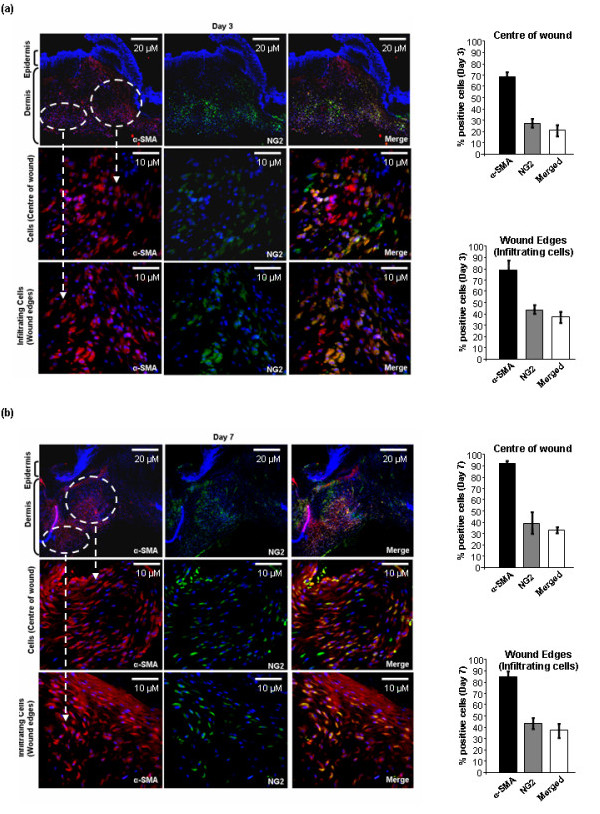
**A subset of the myofibroblasts within wound tissue are pericytes**. Skin of connective tissue growth factor/enhanced green fluorescent protein (CTGF/E-GFP) hemizygous mice post-wounding (a) day 3 and (b) day 7 was fixed, sectioned, and stained with 4',6-diamidino-2-phenylindole (DAPI) to detect nuclei, anti-α-smooth muscle actin (SMA) antibody to detect myofibroblasts, and anti-NG2 antibody to detect pericytes (10 × magnification of dermal tissue). The center of the wound and wound edges were examined and the percentage of fibroblasts within the wound that were α-SMA positive, NG2 positive, and both NG2 and α-SMA positive was calculated as described in Methods. At day 3, note that ~20% of the cells in the wound area were both α-SMA and NG2 positive whereas ~40% of the cells at the wound edges were α-SMA and NG2 positive, indicating that pericytes are recruited to the wound from surrounding tissue. At day 7, note that essentially all cells in the wound area are myofibroblasts. Approximately 30% of the cells in the center of the wound are myofibroblasts of pericyte origin. In the wound edge, ~40% of the cells are myofibroblasts of pericyte origin. Representative data from four wounds from four separate animals per timepoint is shown.

A large body of evidence indicates that vascular pericytes may be a major source of activated myofibroblasts during normal wound healing and fibrosis [4,5]. To investigate (a) the precise contribution of pericytes to the activated fibroblasts present in wounded tissue, and (b) if the CTGF promoter was active in pericytes, we first performed double immunolabeling of tissue sections with anti-NG2 antibody, to detect pericytes, and anti-α-SMA antibody, to address the relative contribution of pericytes to the activated myofibroblasts in the wound. At day 3 post-wounding, 70–80% of the cells in the wound, whether at the edge or in the center, were myofibroblasts, as detected by anti-α-SMA antibody (Figure 4a). Of these, ~30–40% stained positive with an anti-NG2 antibody (Figure 3a). At day 7 post-wounding, ~90% of the cells were myofibroblasts (Figure 4b). At this timepoint, ~40% were NG2 positive; overall approximately a third of the myofibroblasts were also pericytes (Figure 4b).

### The CTGF promoter is active in pericytes

**Figure 5:**
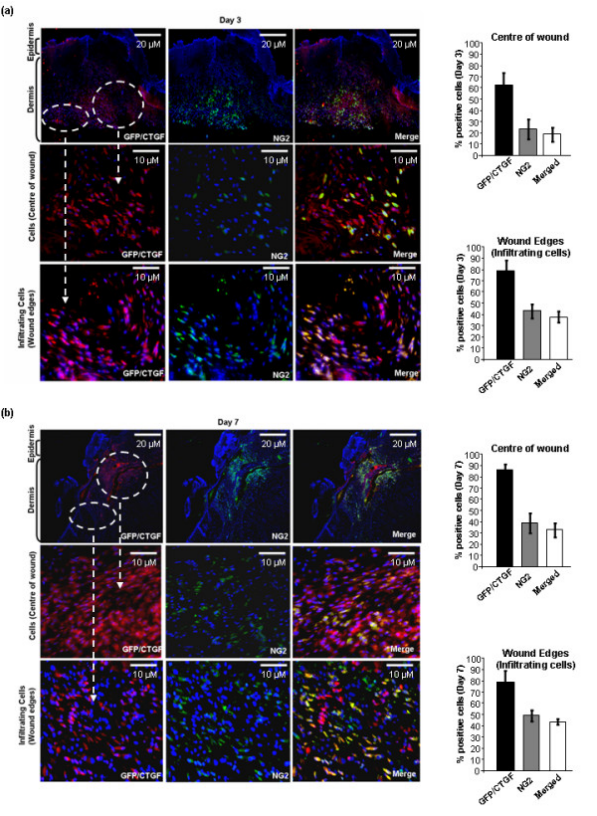
**The connective tissue growth factor (CTGF) promoter is active in pericytes**. Skin of CTGF/enhanced green fluorescent protein (E-GFP) hemizygous mice post-wounding (a) day 3 and (b) day 7 was fixed, sectioned, and stained with 4',6-diamidino-2-phenylindole (DAPI) to detect nuclei, anti-GFP antibody to detect cells in which the CTGF promoter is active, and anti-NG2 antibody to detect pericytes (10 × magnification of dermal tissue). The center of the wound and wound edges were examined and the percentage of fibroblasts within the wound that were GFP positive, NG2 positive, and both NG2 and GFP positive was calculated as described in Methods. At day 3 and 7, note that essentially all pericytes were GFP positive, and hence showed CTGF promoter activity. Representative data from four wounds from four separate animals per timepoint is shown.

To investigate whether the CTGF promoter was active in pericytes, we stained sections with anti-GFP and anti-NG2 antibodies. These data revealed that, at 3 days post-wounding, ~60% of the cells at the center of the wound and ~80% of the cells at the wound edge were GFP positive while ~20% and ~40%, respectively, of the cells were both GFP and NG2 positive (Figure 5a). By day 7 post-wounding ~80% of the cells at the center and wound edge were GFP positive, whereas ~30% of the cells at the center of the wound and ~45% at the wound edge were both GFP and NG2 positive (Figure 5b). These results indicate that the CTGF promoter is active in pericytes. Collectively, our data strongly indicate that CTGF expression is an excellent marker for activated fibroblasts participating in wound healing *in vivo*.

## Discussion

The CCN family comprises six secreted proteins grouped together on the basis of a similar predicted modular secondary structure [6]. CTGF is induced during tissue repair, and elevated, constitutive CTGF expression is a hallmark of fibrosis [11,13]. Although many studies in cell culture have shown that CTGF expression can be induced in fibroblasts, e.g. by transforming growth factor (TGF)β and endothelin-1 [14,24], CTGF can be induced in other cell types as well [6]. Based on the evidence in the literature, it is unclear whether the CTGF promoter is active in normal skin and whether it is induced in fibroblasts or epithelia post-wounding [11,20]. In this report, we show that CTGF promoter activity is essentially absent in normal skin, but is induced post-wounding in myofibroblasts. Although pericytes are believed to contribute to the total number of myofibroblasts in wound tissue [4,5], for the first time we show that pericytes comprise about a third of the number of activated wound fibroblasts. Consistent with the notion that CTGF is expressed in activated fibroblasts, the CTGF promoter was active in pericytes. Our data support the hypothesis that CTGF promoter activity is a good marker of fibroblast activation and fibrogenesis *in vivo*, and that CTGF may be a key selective contributor to fibrogenesis and tissue repair *in vivo *[25]. Identification of the specific cell types that contribute to recruited CTGF- and α-SMA-expressing myofibroblasts in tissue repair is essential for delineating new mechanisms underlying (myo)fibroblast biology during tissue repair and fibrosis.

## Methods

### Generation of E-GFP tagged mice

Mice were derived from a screen initially conduced by Gong and colleagues [23]. Modified BACs containing inserted E-GFP upstream of targeted genes were injected into pronuclei of FVB/N fertilized mouse oocytes. Hemizygous progeny (STOCK Tg(Ctgf-EGFP)156Gsat; Mutant Mouse Regional Resource Centers (MMRRC [26])) were mated to Swiss Webster mice each generation thereafter. To detect the transgene, mice were subjected to the polymerase chain reaction (PCR) with CACGTAGGAGGATGGCGCAGGGCTAG and TAGCGGCTGAAGCACTGCA as primers, using a standard protocol as described on the MMMRRC website [27].

### Wound surgery

Mice (6 weeks old) containing one copy of the CTGF/E-GFP allele were anesthetized by intraperitoneal injection of 90 μg ketamine plus 10 μg xylazine/g, and their back skin was shaved, depilated with commercially available hair removal cream (Nair; Church and Dwight, Princeton, NJ, USA) and cleaned with alcohol. Using a sterile 4-mm biopsy punch, four bilateral full-thickness skin wounds were created on the dorsorostral back skin. Wounds were separated by a minimum of 6 mm of uninjured skin. Mice were killed by CO_2 _euthanasia after 0, 3 and 7 days post-wounding and wound tissue biopsies were collected for immunoflourescence.

### Immunofluorescence

Wound tissue sections (0.5 μm) were cut using a microtome (Leica, Richmond Hill, ON, Canada) and collected on Superfrost Plus slides (Fisher Scientific, Ottawa, ON, Canada). Sections were then de-waxed in xylene and rehydrated by successive immersion in descending concentrations of alcohol. Sections were then subjected to single or double immunofluorescence. Briefly, tissue sections were incubated with mouse serum for 30 min and washed with phosphate-buffered saline (PBS). Sections were then incubated with primary antibodies for 1 h at room temperature under humidified conditions. Primary antibodies used alone (single immunofluorescence) or in combination (double immunofluorescence) were: rabbit anti-green fluorescent protein (anti-GFP; 1:100 dilution, Invitrogen, **Burlington, ON, Canada**), mouse anti-NG2 (pericyte marker, 1:100 dilution, Chemicon, Millipore, **Billerica, MA, USA)**, mouse anti-alpha-smooth muscle actin (α-SMA, 1:100 dilution, Sigma, St Louis, MO, USA). Double immunofluorescence for α-SMA and NG2 was performed using rabbit polyclonal antibody for α-SMA (Abcam, Cambrodge, MA, USA) and mouse antibody for NG2. Both rabbit and mouse antibodies for α-SMA showed identical staining of cells and tissues in our system. After primary antibody incubation, sections were then washed with PBS and incubated with appropriate fluorescent secondary antibodies (Jackson Immunoresearch, West Grove, PA, USA) for 1 h at room temperature. Sections were then washed with PBS and mounted using 4',6-diamidino-2-phenylindole (DAPI) and photographed using a Zeiss fluorescence microscope and Northern Eclipse software (Empix, Missassagua, ON, Canada). Six independent fields were examined per data point.

The tissue expression of GFP/CTGF and α-SMA on days 0, 3 and 7 post-wounding was graded on a scale of 0–3 by three blinded observers; 0 signifies no staining, 1 signifies very little staining, 2 signifies moderate staining, 3 signifies extensive staining.

Sections undergoing double immunofluorescence were photographed at 10 × (for whole tissue section) and 60 × (for cells) magnifications. To detect number of α-SMA, GFP/CTGF or NG2 positive cells in and around wound area, two planes were chosen: (a) cells in the centre of the wound, and (b) infiltrating cells at wound edges. At each plane, the total number of cells/mm^2 ^were counted. Subsequently, the number of α-SMA, GFP/CTGF and NG2 positive cells/mm^2 ^were counted and expressed as percentage positive cells at each plane.

### Statistical analysis

Statistical analysis was performed using the Student t test. Results are expressed as the mean ± standard error of the mean (SEM). A p value < 0.05 was considered statistically significant.

## Competing interests

The authors declare that they have no competing interests.

## Authors' contributions

AL designed and conceived of the study, MK, SL, KH, SP and LK performed and interpreted the experiments and performed statistical analyses, AL, MK and SL wrote the manuscript.
